# The Relationship between Hypomagnesemia, Metformin Therapy and Cardiovascular Disease Complicating Type 2 Diabetes: The Fremantle Diabetes Study

**DOI:** 10.1371/journal.pone.0074355

**Published:** 2013-09-03

**Authors:** Kirsten E. Peters, S. A. Paul Chubb, Wendy A. Davis, Timothy M. E. Davis

**Affiliations:** 1 University of Western Australia, School of Medicine and Pharmacology, Fremantle Hospital, Fremantle, Western Australia, Australia; 2 Department of Biochemistry, PathWest Laboratory Medicine, Fremantle Hospital, Fremantle, Western Australia, Australia; Mayo Clinic, United States of America

## Abstract

**Background:**

Low serum magnesium concentrations have been associated with cardiovascular disease risk and outcomes in some general population studies but there are no equivalent studies in diabetes. Metformin may have cardiovascular benefits beyond blood glucose lowering in type 2 diabetes but its association with hypomagnesemia appears paradoxical. The aim of this study was to examine relationships between metformin therapy, magnesium homoeostasis and cardiovascular disease in well-characterized type 2 patients from the community.

**Methods and Findings:**

We studied 940 non-insulin-treated patients (mean±SD age 63.4±11.6 years, 49.0% males) from the longitudinal observational Fremantle Diabetes Study Phase I (FDS1) who were followed for 12.3±5.3 years. Baseline serum magnesium was measured using stored sera. Multivariate methods were used to determine associates of prevalent and incident coronary heart disease (CHD) and cerebrovascular disease (CVD) as ascertained from self-report and linked morbidity/mortality databases. 19% of patients were hypomagnesemic (serum magnesium <0.70 mmol/L). Patients on metformin, alone or combined with a sulfonylurea, had lower serum magnesium concentrations than those on diet alone (*P*<0.05). There were no independent associations between serum magnesium or metformin therapy and either CHD or CVD at baseline. Incident CVD, but not CHD, was independently and inversely associated with serum magnesium (hazard ratio (95% CI) 0.28 (0.11–0.74); *P = *0.010), but metformin therapy was not a significant variable in these models.

**Conclusions:**

Since hypomagnesemia appears to be an independent risk factor for CVD complicating type 2 diabetes, the value of replacement therapy should be investigated further, especially in patients at high CVD risk.

## Introduction

It has been recognized for more than 50 years that low serum magnesium concentrations can be found in patients with diabetes [1], with a prevalence of hypomagnesemia in a number of previously-published studies of between 25% and 38% [2]–[4]. This association may reflect a ‘vicious cycle’ with hyperinsulinemia associated with insulin resistance contributing to extracellular magnesium depletion and, in turn, further augmentation of insulin resistance by hypomagnesemia [5], [6]. Low serum magnesium concentrations are also associated with dyslipidemia, hypertension, endothelial dysfunction and inflammation, and thus the development of cardiovascular disease [5], [7], [8] including in diabetes [6].

Metformin is widely recommended as first-line treatment for type 2 diabetes from the time of diagnosis [9]. Although the evidence from pooled randomized trials has been questioned [10], [11], this recommendation reflects the results of the UK Prospective Diabetes Study (UKPDS), the longest and amongst the largest such trials to date, which showed metformin to have favorable effects on cardiovascular disease independent of blood glucose lowering [12], [13]. If metformin has cardiovascular benefit, or even if its effect is neutral [10], [11], this would appear inconsistent with evidence from human studies that metformin reduces serum magnesium [2], [4], [14], [15] and maintains diabetes-associated hypomagnesemia [3], [16], effects that would theoretically increase vascular risk [5]–[7]. The influence of metformin on magnesium homoeostasis is, however, complex, with evidence of an increase in intracellular magnesium [16], [17] that may offset the potential adverse effects of hypomagnesemia. In addition, there may be other major influences on magnesium metabolism in diabetic patients, including renal disease, gastrointestinal disorders, and a variety of other drugs that lower serum magnesium including alcohol, diuretics and proton pump inhibitors [18].

Since there have been no studies of the relationship between metformin therapy, magnesium homoeostasis and cardiovascular disease in type 2 diabetes, we have measured serum magnesium and its fractional excretion in well-characterized community-based patients with type 2 diabetes followed for up to 18 years for incident cardiovascular events. We examined the prevalence and associates of hypomagnesemia, and whether serum magnesium is independently associated with prevalent and incident coronary heart disease (CHD) and cerebrovascular disease (CVD), the two major macrovascular complications of type 2 diabetes. Based on the available evidence, our principal hypothesis was that metformin is associated with hypomagnesemia but that this does not have adverse cardiovascular effects.

## Methods

### Ethics Statement

The Fremantle Diabetes Study Phase I (FDS1) protocol was approved by the Human Rights Committee at Fremantle Hospital, Fremantle, Western Australia, and all subjects gave written informed consent before participation.

### Patients

The FDS1 is a prospective longitudinal observational study of patients with diabetes living in the urban primary catchment area of Fremantle Hospital in the state of Western Australia, a population of approximately 120,000. A full description of the FDS1 cohort, as well as of non-recruited patients, has been published elsewhere [19]. Of 1,296 patients with type 2 diabetes recruited between 1993 and 1996, 940 were eligible for inclusion in the present sub-study, based on availability of serum and/or urine for assay of magnesium concentrations and, because insulin therapy may affect serum magnesium [5], [20], non-insulin treatment at baseline.

### Clinical and Laboratory Assessment

Detailed baseline questionnaire, clinical, biochemical and other diabetes-related data were collected at study entry and each annual review [19]. This included history relevant to magnesium homoeostasis including a full list of prescribed and over-the-counter medications including magnesium supplementation, and gastrointestinal problems associated with malabsorption such as inflammatory bowel disease, coeliac disease and diverticulitis. At each visit, fasting blood and first-morning urine samples were taken and routine-care biochemical assays were performed promptly using standard automated methods in a single nationally accredited laboratory. Additional serum, plasma and urine samples were stored at −80°C for subsequent analyses.

In the present sub-study, serum and/or urinary magnesium concentrations were measured using stored baseline samples. Magnesium was estimated using an arsenazo dye based method on an Architect ci8200 analyser (Abbott Diagnostics, Doncaster, Victoria, Australia) using reagents supplied by the manufacturer. Urine samples were diluted 1∶5 prior to assay. Between-day imprecision was 6.3% and 3.2% at 0.5 mmol/L and 1.8 mmol/L serum magnesium, respectively, and 6.9% and 4.1% at 2.0 mmol/L and 4.8 mmol/L urine magnesium, respectively. Hypomagnesemia was defined as serum magnesium <0.70 mmol/L. The urinary fractional excretion of magnesium (FEMg) was defined as [magnesium (urine)×creatinine (serum)×100]/[magnesium (serum)×creatinine (urine)], with all units in mmol/L and FEMg itself as a percentage [21]. Normomagnesemic control subjects have an FEMg of 0.5–4.0%, hypomagnesemic patients with extra-renal Mg loss have FEMg of <2.7%, and those with renal Mg loss have FEMg>4.0% [22].

### Cardiovascular Disease Ascertainment

Longitudinal outcome data were obtained from self-report and linkage of the FDS database to the Western Australia Data Linkage System (WADLS) [23] which includes all public and private hospitalizations, and the state death register. Using FDS and WADLS databases, comprehensive details of cardiovascular disease events, both fatal and non-fatal, were available from 1982 until end-2010, a follow-up from FDS1 study entry of 11,593 patient-years (12.3±5.3 years). Patients were classified as having prevalent coronary heart disease (CHD) if there was a self-reported history/documented prior hospital admission for myocardial infarction (MI), angina, or revascularization. Prevalent cerebrovascular disease (CVD) was assessed from self-report/prior hospital admissions for stroke and/or transient attack. Incident CHD and CVD events were ascertained from self-report and WADLS documentation. Other chronic complications of diabetes were determined using standard criteria [24].

### Statistical Analysis

All data analyses were performed in the computer package SPSS for Windows (version 19). Variables were natural log (ln) transformed where appropriate. Data are presented as proportions, mean±SD, geometric mean (SD range), or, in the case of variables which did not conform to a normal or log-normal distribution, median [inter-quartile range, IQR]. For independent samples, two-way comparisons for proportions were by Fisher’s exact test, for normally distributed variables by Student’s *t*-test, and for non-normally distributed variables by Mann-Whitney U-test. A two-tailed significance level of *P*<0.05 was used throughout.

Multiple linear regression analysis (stepwise variable selection with *P*<0.05 for entry and >0.10 for removal) was used to determine baseline associates of serum magnesium. Multiple logistic regression (forward conditional variable selection with *P*<0.05 for entry and >0.10 for removal) was used to investigate independent associates of prevalent CHD and CVD. Cox proportional hazards modelling (forward conditional variable selection with *P*<0.05 for entry and >0.10 for removal) with age as the time-line was used to determine independent baseline predictors of incident CHD and CVD and composite CHD/CVD. All clinically plausible variables with bivariate *P*≤0.20 were considered for entry into these models in a forward stepwise manner and included demographic and diabetes-related factors, the presence of other complications and cardiovascular risk factors. After the most parsimonious model in each instance was defined, serum magnesium and metformin use were entered.

## Results

### Baseline Patient Characteristics

At study entry, the 940 patients eligible for the present study did not differ significantly from the 194 remaining type 2 FDS participants not taking insulin in body mass index (BMI) (29.5±5.4 vs 30.1±6.0 kg/m^2^; *P* = 0.18), gender (49.0% vs 47.4% males; *P* = 0.69), diabetes duration (3.0 [0.8–6.0] vs 4.0 [1.0–7.0] years; *P* = 0.16) or HbA_1c_ (7.3 [6.3–8.6] vs 7.3 [6.4–8.5]% or 56 [45–70] vs 56 [46–69] mmol/mol; *P* = 0.91), but were significantly younger in age (63.4±11.6 vs 65.3±10.9 years; *P* = 0.032).

Table 1 summarizes the differences between patients by type of blood glucose-lowering therapy at baseline. Patients on metformin, whether alone or in combination with a sulfonylurea, had significantly lower serum magnesium concentrations (and a greater percentage were hypomagnesemic) than those on diet alone. The same pattern was in evidence for FEMg. There were no significant-between group differences in other factors that could alter magnesium metabolism such as renal function and other therapies.

**Table 1:** Baseline characteristics of patients by blood glucose-lowering therapy including variables relevant to magnesium homoeostasis. Data are percentages, mean±SD, geometric mean (SD range) or median [inter-quartile range]. ^a^<0.05 vs diet alone; ^b^<0.05 vs metformin alone; ^c^<0.05 vs sulfonylurea alone, unadjusted for multiple comparisons.

	Diet	Metformin only	Sulfonylurea only	Metformin+Sulfonylurea	*P-*value
Number	350	148	258	184	
Age (years)	62.7±11.7	60.5±12.2	65.9±10.9 a,b,	63.4±11.4^ b,c^	<0.001
Sex (% male)	48.6	43.9	53.9	47.3	0.24
Diabetes duration (years)	1.0 [0.3–3.0]	3.0 [0.8–5.0]^a^	4.0 [1.3–7.0]a,b	8.0 [5.0–12.0]a,b,c	<0.001
Body mass index (kg/m^2^):	29.2±5.7	31.2±5.2^a^	28.7±5.3^b^	29.9±5.0^ b,c^	<0.001
Waist circumference (cm):					
Men	100.7±11.1	107.9±10.4^a^	101.6±11.9^b^	104.8±11.9a,c	<0.001
Women	96.2±14.0	98.2±11.5	94.6±12.7	98.5±11.9	0.09
Fasting serum glucose (mmol/L)	7.2 [6.2–8.6]	8.7 [7.1–10.2]^a^	8.7 [7.4–11.1]^a^	9.9 [8.0–11.9]a,b,c	<0.001
HbA_1c_ (%)	6.5 [5.9–7.4]	7.6 [6.7–8.7]^a^	7.9 [6.6–8.9]^a^	8.3 [7.1–9.9]a,b,c	<0.001
Serum magnesium (mmol/L)	0.85±0.16	0.81±0.15^a^	0.83±0.14	0.74±0.17a,b,c	<0.001
Hypomagnesemia (%)	11.1	17.6	14.7	41.3 a,b,c	<0.001
Urinary magnesium (mmol/L)	1.80 (1.01–3.20)	1.46 (0.84–2.54)^a^	1.63 (0.89–2.97)^a^	1.31 (0.73–2.37) a,c	<0.001
Fractional excretion urinary magnesium (%)	2.2 (1.1–4.4)	1.8 (0.9–3.6)^a^	2.1 (1.1–4.0)^b^	1.9 (0.9–4.0)^a^	0.006
Renal causes (>4.0%) (%)	15.7	10.4	14.1	12.5	0.13
Serum creatinine (mmol/L)	95±75	86±30	93±57	88±55	0.38
Alcohol consumption (standard drinks/day)	0.0 [0.0–0.8]	0.0 [0.0–0.3]	0.0 [0.0–0.5]	0.0 [0.0–0.3]	0.64
Diuretic therapy (%)	18.3	22.3	26.4	17.9	0.07
Digoxin therapy (%)	6.9	7.4	7.4	3.3	0.24
Proton pump inhibitor therapy (%)	0.6	1.4	0.4	1.1	0.58
Magnesium supplementation (n = 2)(%)	0.4	0	0	0.7	0.80
Gastrointestinal problems (%)	2.9	2.0	1.6	3.3	0.64

Data are percentages, mean ± SD, geometric mean (SD range) or median [inter-quartile range].

a *P*<0.05 vs diet alone; ^b^ *P*<0.05 vs metformin alone; ^c^ *P*<0.05 vs sulfonylurea alone, unadjusted for multiple comparisons.

Table 2 summarizes baseline characteristics in the 761 (81%) normo- and 179 (19%) hypomagnesemic patients. Those with hypomagnesemia had longer diabetes duration, higher fasting serum glucose and HbA_1c_, lower ß-cell function, greater prevalence of obesity (BMI ≥30.0 kg/m^2^), larger waist circumference in males, higher waist-hip-ratio in females, higher systolic and diastolic blood pressures, greater use of blood pressure-lowering medications (especially diuretics), higher urinary albumin:creatinine ratio, and exercised less compared to people with normal serum magnesium levels. FEMg was significantly lower in the normomagnesemic group. There were similar numbers of patients with gastrointestinal problems in the two groups. Only two people were taking magnesium supplementation and both were normomagnesemic.

**Table 2:** Baseline characteristics of type 2 patients by serum magnesium category. Data are percentages, mean±SD, geometric mean (SD range) or median [inter-quartile range].

	Normomagnesemia	Hypomagnesemia	*P-*value
Number (%)	761 (81)	179 (19)	
Serum magnesium (mmol/L)	0.87±0.12	0.58±0.09	
Fractional excretion urinary magnesium (%)	1.9 (1.0–3.8)	2.7 (1.4–5.3)	<0.001
Age (years)	63.2±11.5	64.2±12.1	0.29
Sex (% male)	48.6	50.8	0.62
Ethnic background (% Anglo-Celt)	62.0	65.4	0.60
Smoking status (% Never/Ex/Current)	44.6/41.1/14.3	45.3/39.7/15.1	0.92
Any exercise in past two weeks (%)	75.0	61.5	<0.001
Alcohol consumption (standard drinks/day)	0.0 [0.0–0.4]	0.0 [0.0–0.8]	0.50
Age at diabetes diagnosis (years)	58.5±11.8	58.3±12.8	0.85
Diabetes duration (years)	3.0 [0.8–6.0]	4.8 [1.0–8.0]	0.010
Diabetes treatment (%)			
Diet	40.9	21.8	<0.001
Metformin only	16.0	14.5	
Sulfonylurea only	28.9	21.2	
Metformin plus sulfonylurea	14.2	42.5	
Fasting serum glucose (mmol/L)	8.1 [6.7–10.3]	8.9 [7.2–11.1]	0.003
HbA_1c_ (%)	7.2 [6.3–8.5]	7.5 [6.6–8.9]	0.019
Fasting serum insulin (pmol/L)	75.3 (38.9–146.9)	76.0 (38.1–151.5)	0.88
Beta cell function (HOMA2B; % normal)	45.2 (22.4–91.4)	40.2 (18.7–86.1)	0.05
Insulin sensitivity (HOMA2S; % normal)	62.9 (32.4–122.1)	60.6 (30.2–121.3)	0.50
BMI (kg/m^2^) (%):	29.4±5.4	30.2±5.2	0.07
Waist circumference (cm):			
Men	102.2±11.3	105.1±12.8	0.050
Women	96.2±13.0	98.4±12.5	0.16
Waist:hip ratio:			
Men	0.95±0.05	0.96±0.06	0.21
Women	0.86±0.06	0.88±0.07	0.015
Systolic blood pressure (mmHg)	149±23	153±23	0.033
Diastolic blood pressure (mmHg)	80±11	83±12	0.002
Taking antihypertensive medication (%)	49.0	57.5	0.046
ACE-inhibitor	20.9	19.6	0.76
Beta-blocker	16.3	17.9	0.66
Calcium channel blocker	20.6	19.6	0.84
Diuretic	19.3	28.5	0.008
Total serum cholesterol (mmol/L)	5.5±1.1	5.4±1.1	0.08
Serum HDL-cholesterol (mmol/L)	1.05±0.32	1.03±0.31	0.57
Serum LDL-cholesterol (mmol/L)	3.5±0.9	3.4±0.9	0.10
Serum triglycerides (mmol/L)	1.9 (1.1–3.3)	2.0 (1.1–3.5)	0.53
Taking lipid-lowering medication (%)	10.8	9.0	0.59
Serum creatinine (µmol/L)	92±64	89±43	0.48
Urinary albumin:creatinine ratio (mg/mmol)	2.6 (0.7–10.6)	3.8 (0.8–17.3)	0.004
Peripheral sensory neuropathy (%)	26.9	33.7	0.09
Any retinopathy (%)	11.1	14.0	0.29
Coronary heart disease (%)	26.7	29.6	0.46
Cerebrovascular disease (%)	8.9	10.1	0.67
Peripheral arterial disease (%)	28.2	22.0	0.11
Proton pump inhibitor use (%)	0.8	0.6	1.00
Gastrointestinal problems (%)	2.6	1.7	0.60

Data are percentages, mean±SD, geometric mean (SD range) or median [inter-quartile range].

### Baseline Associates of Serum Magnesium

In the most parsimonious multivariate model, serum magnesium as a continuous variable was independently associated with total serum cholesterol and negatively with BMI, fasting serum glucose and systolic blood pressure (see Table 3). After adjusting for these variables, metformin monotherapy was of borderline significance (*P* = 0.052), but sulfonylurea therapy and metformin in combination with a sulfonylurea were independently associated with a lower serum magnesium.

**Table 3:** Independent associates of serum magnesium at baseline in the most parsimonious model (adjusted R^2^ = 0.136), with entry of blood glucose-lowering treatment group after adjustment for these variables.

	Regression coefficient, ß (95% CI)	*P*-value
(Constant)	0.944 (0.846, 1.041)	<0.001
Fasting serum glucose (per 1 mmol/L)	−0.004 (−0.007, 0.000)	0.030
BMI (per 1 kg/m^2^)	−0.004 (−0.006, −0.002)	<0.001
Diastolic blood pressure (per 10 mmHg)	−0.001 (−0.002, 0.000)	0.081
Total cholesterol (mmol/L)	0.026 (0.017, 0.034)	<0.001
Fractional excretion of Mg (%)	−0.011 (−0.015, −0.007)	<0.001
After adjustment (diet-treated as reference):		
On metformin only	−0.030 (−0.059, 0.000)	0.052
On sulfonylurea only	−0.026 (−0.051, −0.001)	0.041
On both metformin and sulfonylurea	−0.105 (−0.134, −0.077)	<0.001

The geometric mean (SD range) FEMg was significantly lower in both the metformin monotherapy group (1.8 (0.9–3.6)%) and those on combination metformin-sulfonylurea (1.9 (0.9–4.0)%) compared with those on diet alone (2.2 (1.1–4.4)%; *P*<0.05 in both cases) but sulfonylurea monotherapy patients had a FEMg similar to that in the diet alone group (2.1 (1.1–4.0)%).

### Prevalent Coronary Heart Disease and Cerebrovascular Disease

The independent associates of prevalent CHD and CVD at baseline in the most parsimonious models are shown in Table 4. After adjustment for these risk factors, neither metformin use (either alone or in combination with a sulfonylurea) nor serum magnesium concentration were significant associates in either model (*P*≥0.13 in each case).

**Table 4:** Independent associates of prevalent coronary heart disease (CHD) and cerebrovascular disease (CVD) at baseline.

	CHD odds ratio		CVD odds ratio	
	(95% CI)	*P*-value	(95% CI)	*P*-value
Age (for a 10-year increase)	1.34 (1.14–1.57)	<0.001	1.94 (1.50–2.50)	<0.001
Male gender	1.74 (1.22–2.48)	0.002		
Ex-smoker	1.42 (1.01–2.02)	0.047	1.72 (1.03–2.88)	0.040
Current smoking			2.66 (1.32–5.34)	0.006
Any exercise in past two weeks	0.54 (0.38–0.76)	0.001		
Taking antihypertensive medications	3.77 (2.65–5.38)	<0.001	2.20 (1.31–3.67)	0.003
Taking lipid-modifying medications	3.32 (2.06–5.35)	<0.001		
Taking digoxin	2.17 (1.21–3.89)	0.009		

### Incident Coronary Heart Disease and Cerebrovascular Disease

There were 254 patients who had a new CHD event and 175 had a new CVD event during follow-up. The independent associates of incident CHD, CVD and combined CHD/CVD in the most parsimonious models are shown in Table 5. After adjustment for these risk factors, metformin use (either alone or in combination with a sulfonylurea) was not a significant associate in any of the three models (*P*≥0.25 in each case) but incident CVD and combined CHD/CVD were independently and inversely associated with serum magnesium (*P≤*0.011 in each case). The interaction term serum magnesium*metformin (yes/no) was not a significant predictor in any of the three models after adjustment for significant variables (*P*≥0.37).

**Table 5:** Hazard ratios (HR) and 95% confidence intervals (95% CI) from the most parsimonious Cox models for incident coronary heart disease (CHD), incident cerebrovascular disease (CVD), and combined incident CHD/CVD using age at census as the timeline, with metformin use and serum magnesium concentration added to the models.

	CHD	*P-*value	CVD	*P-*value	CHD/CVD	*P-*value
	HR (95% CI)		HR (95% CI)		HR (95% CI)	
Age at diabetes diagnosis (5-year increase)	0.71 (0.66–0.76)	<0.001	0.77 (0.70–0.84)	<0.001	0.74 (0.70–0.79)	<0.001
Male gender	1.43 (1.11–1.85)	0.005			1.59 (1.24–2.04)	<0.001
Current smoking habit	1.78 (1.28–2.48)	0.001			1.46 (1.05–2.03)	0.023
Total serum cholesterol (1 mmol/L increase)					1.16 (1.04–1.30)	0.009
Ln(serum triglycerides (mmol/L))*	1.37 (1.10–1.72)	0.005				
Systolic blood pressure (10 mmHg increase)			1.12 (1.05–1.19)	0.001		
Ln(urinary albumin:creatinine)*					1.12 (1.03–1.21)	0.006
Atrial fibrillation			2.78 (1.74–4.43)	<0.001		
Taking metformin	0.84 (0.63–1.12)	0.25	0.99 (0.71–1.40)	0.99	0.87 (0.66–1.13)	0.29
Serum magnesium (1 mmol/L increase)	0.52 (0.23–1.18)	0.12	0.28 (0.11–0.74)	0.010	0.39 (0.19–0.80)	0.011

* A 2.72 fold increase in serum triglycerides or urinary albumin:creatinine corresponds to an increase of 1 in the natural logarithm of each variable.

## Discussion

The present study shows that hypomagnesemia occurs in 1 in 5 community-based non-insulin-treated patients with type 2 diabetes. Oral hypoglycaemic therapy, especially metformin in combination with sulfonylurea therapy, is associated with low serum magnesium concentrations. A relatively low FEMg in the metformin-treated patients suggests renal conservation in the presence of reduced gastrointestinal absorption and/or increased intracellular accumulation. Consistent with its known associations with cardiovascular risk factors, endothelial dysfunction and inflammation [5], a low baseline serum magnesium proved an independent predictor of subsequent CVD and CHD/CVD, but not CHD on its own, in FDS1 patients during long-term follow-up. Metformin treatment itself was not associated with these cardiovascular outcomes.

The prevalence of hypomagnesemia in previously-published studies of diabetic patients ranges from 25% to 38% [2]–[4], higher than the 19% in the present study. Our data are difficult to compare with these results because the authors did not report factors such as renal function and concomitant drug therapy [18] that may have modified serum magnesium concentrations. Notwithstanding these potential between-study differences, it is possible that a relatively high dietary magnesium intake reduced the risk of hypomagnesemia in our patients. Indeed, the hardness of the water supply in the state of WA [25] coupled with the observation that a litre of hard water can contribute around half of the daily recommended magnesium intake [26] could mean that our patients enjoyed relative protection against hypomagnesemia through the local water supply relative to that in other geographic locations. Expected bivariate associates of hypomagnesemia [5], including with hyperglycemia and high blood pressure, were present in our data and these variables were also independent predictors in multivariate modelling.

The suggestion that biguanide treatment was associated with hypomagnesemia was first made by Mather and coworkers [2]. McBain *et al.* [3] subsequently observed that, in a small sample of 34 diabetic patients starting oral hypoglycemic therapy, metformin did not improve the hypomagnesemia of diabetes but did reduce urinary magnesium output. In contrast, patients starting sulfonylurea therapy experienced improvement of serum magnesium with no change in urinary clearance [3]. A case report in which metformin treatment caused symptomatic severe hypomagnesemia (0.33 mmol/L) following several months of worsening diarrhea, both of which improved when metformin was stopped, suggests that metformin-associated gastrointestinal magnesium loss may contribute to hypomagnesemia [14].

Our results parallel those of McBain *et al*. [3]. Those patients taking metformin with or without a sulfonylurea had lower FEMg than the patients on either diet or sulfonylurea alone, similar to the smaller sample studied by McBain *et al* [3]. This suggests that there is reduced renal magnesium output that offsets increased gastrointestinal loss [14] and/or increased uptake by tissues such as erythrocytes [16] and peripheral blood monocytes [17]. Given that the most profound hypomagnesemia was seen in our patients on combination metformin-sulfonylurea whose median diabetes duration was at least double that in the other three treatment groups, and the bivariate association between hypomagnesemia and diabetes duration in the total cohort, our data are consistent with the possibility that, as with serum vitamin B_12_ concentrations [27], the risk of hypomagnesemia due to gastrointestinal loss increases with duration of metformin therapy. By contrast, those patients on diet alone with short duration of diabetes and relatively good glycemic control are at the lowest risk of hypomagnesemia.

Our study showed that serum magnesium was a significant independent predictor of incident CVD but not incident CHD. The magnitude of this effect was a 6% reduction in risk per 0.1 mmol/L increase in serum magnesium. General population studies of the association between hypomagnesemia and ischemic stroke have had mixed results. In a study of 323 patients at increased risk due to peripheral arterial disease who were followed for a median of 20 months, those in the lowest tertile of serum magnesium had a significant 3.3-fold increased risk compared to those in the highest tertile after adjustment for other vascular risk factors [28]. By contrast, the Atherosclerosis Risk in the Community (ARIC) study showed no association between serum magnesium and incident ischemic stroke after adjustment for hypertension and diabetes over 15 years of follow-up [29]. These authors found that hypomagnesemia was associated with both hypertension and diabetes, and that these latter parameters accounted for nearly all the increased stroke risk. The reasons for the discrepancy between these two studies are unclear but may relate to the large differences in baseline cardiovascular risk and duration of follow-up. Low dietary intake of magnesium is, however, associated with incident ischemic stroke, as shown by a meta-analysis of seven prospective studies [30], while randomized acute post-stroke magnesium replacement trials are in progress [31] (NCT00059332 and NCT01502761).

General population studies of the association between serum magnesium and incident CHD have also yielded mixed results. In a northern German population-based sample, a serum magnesium <0.73 mmol/L was a significant independent predictor of all-cause and cardiovascular mortality after adjustment for cardiovascular risk factors including diabetes and hypertension [32]. By contrast, hypomagnesemia was not a significant predictor of cardiovascular events, or all-cause or sudden cardiac mortality, in the Framingham Offspring Study [33]. In the ARIC study, incident CHD decreased across quartiles of serum magnesium in partially adjusted analyses in women only, but inclusion of diabetes and hypertension attenuated the relative risks to borderline significance [34]. In our multivariate analyses of incident events, the hazard ratio suggested a 48% reduction in CHD risk but the confidence intervals indicated that this could range from a 77% reduction to an 18% excess.

As we have reported previously [35] and consistent with published meta-analyses [10], [11], metformin therapy was not a significant predictor of either CVD or CHD in FDS1 patients in the present analyses, suggesting that its propensity to cause hypomagnesemia does not modulate cardiovascular risk in diabetic patients. This is perhaps because intracellular magnesium is not reduced [16], [17]. Other significant independent associates of prevalent and incident CVD and CHD were as expected and included age, male sex, smoking, hypertension and dyslipidemia. The statistical models of risk factors for prevalent CVD and CHD included antihypertensive and lipid-lowering therapies which are very likely to represent confounding by indication. Because of this and the more objective nature of prospectively collected endpoints, the incident analyses appear more robust.

The present study had limitations. We did not measure serum magnesium during follow-up. Its longitudinal relationships with outcomes may have been different had these data been available but other factors such as initiation of cardiovascular risk reducing therapies such as statins would also have to be included in complex time-dependent analyses which are seldom performed in observational studies. We did not have baseline measures of intracellular magnesium which have, together with serum concentrations and FEMg, been recommended in the detailed characterization of magnesium homoeostasis in human studies [36]. Magnesium is protein bound, approximately 25% to albumin [37]. We did not have serum albumin concentrations in the present study but only 8 of 1,065 community-based non-insulin-treated type 2 diabetic patients (0.8%) in the later Phase II of the FDS [19] were hypoalbuminemic, and there was no difference in serum albumin in those on diet alone, metformin alone, sulfonylurea alone or metformin plus sulfonylurea (*P* = 0.21 by ANOVA). The strengths of the present study include the prospective design, large numbers of representative community-based patients, detailed baseline assessment, and capture of endpoints through a validated data linkage system.

The present FDS1 sub-study has shown that metformin treatment alone or in combination with a sulfonylurea is associated with hypomagnesemia, especially in those on long-duration therapy. Hypomagnesemia, but not metformin therapy, increases the risk of incident CVD in type 2 diabetes. Since serum magnesium is easily and inexpensively measured, and as oral magnesium replacement is cheap and safe [38], there is an argument for screening of diabetic patients for hypomagnesemia and institution of supplementation if it is detected, especially in those with other risk factors for CVD such as atrial fibrillation and hypertension. This strategy could be assessed in a prospective randomized trial as part of the increasing interest in magnesium as a macronutrient with therapeutic potential [31].

## Supporting Information

STROBE Checklist S1 Detailed STrengthening the Reporting of OBservational studies in Epidemiology (STROBE) requirements for the present cross-sectional case-control observational study.
(DOC)
