# Supplementary material for: The Relationship between Hypomagnesemia, Metformin Therapy and Cardiovascular Disease Complicating Type 2 Diabetes: The Fremantle Diabetes Study
Source: PLoS One. 2013 Sep 3;8(9):e74355. doi: 10.1371/journal.pone.0074355 (PMC3760872; doi:10.1371/journal.pone.0074355)
Supplement: STROBE Checklist S1 — Detailed STrengthening the Reporting of OBservational studies in Epidemiology (STROBE) requirements for the present cross-sectional case-control observational study. (DOC) [file pone.0074355.s001.doc]

STROBE checklist of items that should be included in reports of cross-sectional case-control observational studies with detailed referencing of requirements to the text of the paper.

|  | | Item No | Recommendation |
| --- | --- | --- | --- |
| **Title and abstract** | | 1 | (*a*) Indicate the study’s design with a commonly used term in the title or the abstract  Longitudinal cohort study as stated in the Abstract on page 2 and Patients and Methods on page 4. |
|  |  |  | (*b*) Provide in the abstract an informative and balanced summary of what was done and what was found  Provided in Abstract on page 2. |
| Introduction | | | |
| Background/rationale | | 2 | Explain the scientific background and rationale for the investigation being reported  Included in the Introduction on pages 3 and 4. |
| Objectives | | 3 | State specific objectives, including any prespecified hypotheses  Included in the Introduction on page 4. |
| Methods | | | |
| Study design | | 4 | Present key elements of study design early in the paper  Included in the Patients and Methods on page 4. |
| Setting | | 5 | Describe the setting, locations, and relevant dates, including periods of recruitment, exposure, follow-up, and data collection  Included in the Methods on pages 5, 6 and 7 |
| Participants | | 6 | (*a*) *Cohort study*—Give the eligibility criteria, and the sources and methods of selection of participants. Describe methods of follow-up  *Case-control study*—Give the eligibility criteria, and the sources and methods of case ascertainment and control selection. Give the rationale for the choice of cases and controls  *Cross-sectional study*—Give the eligibility criteria, and the sources and methods of selection of participants  Included in the Patients and Methods on pages 4, 5 and 6. |
|  |  |  | (*b*) *Cohort study*—For matched studies, give matching criteria and number of exposed and unexposed  *Case-control study*—For matched studies, give matching criteria and the number of controls per case  Not applicable. |
| Variables | | 7 | Clearly define all outcomes, exposures, predictors, potential confounders, and effect modifiers. Give diagnostic criteria, if applicable  Included in the Patients and Methods on pages 4, 5 and 6. |
| Data sources/ measurement | | 8* | For each variable of interest, give sources of data and details of methods of assessment (measurement). Describe comparability of assessment methods if there is more than one group  Included in the Patients and Methods on pages 4 and 5. |
| Bias | | 9 | Describe any efforts to address potential sources of bias  Addressed in the limitations paragraph starting at the bottom of page 12/top of page 13 in the Discussion. |
| Study size | | 10 | Explain how the study size was arrived at  Included in the Methods on page 4 (sample was derived from a fixed geographical catchment area) |
| Quantitative variables | | 11 | Explain how quantitative variables were handled in the analyses. If applicable, describe which groupings were chosen and why  Included in the Patients and Methods on pages 5, 6 and 7. |
| Statistical methods | | 12 | (*a*) Describe all statistical methods, including those used to control for confounding  Included in the Patients and Methods on pages 5, 6 and 7. |
|  |  |  | (*b*) Describe any methods used to examine subgroups and interactions  Included in the Patients and Methods on pages 5, 6 and 7. |
|  |  |  | (*c*) Explain how missing data were addressed  Included in the Patients and Methods on pages 4, 5, 6 and 7. |
|  |  |  | (*d*) *Cohort study*—If applicable, explain how loss to follow-up was addressed  *Case-control study*—If applicable, explain how matching of cases and controls was addressed  *Cross-sectional study*—If applicable, describe analytical methods taking account of sampling strategy Included in the Patients and Methods on pages 5, 6 and 7. |
|  |  |  | (*e*) Describe any sensitivity analyses Not applicable. |
| Results | | | |
| Participants | 13* | (a) Report numbers of individuals at each stage of study—eg numbers potentially eligible, examined for eligibility, confirmed eligible, included in the study, completing follow-up, and analysed  Included in the Results on pages 7 and 8. | |
|  |  | (b) Give reasons for non-participation at each stage  Not applicable. | |
|  |  | (c) Consider use of a flow diagram  Not required. | |
| Descriptive data | 14* | (a) Give characteristics of study participants (eg demographic, clinical, social) and information on exposures and potential confounders  Included in the Results on page 7. | |
|  |  | (b) Indicate number of participants with missing data for each variable of interest  Included in the Results on page 7. | |
|  |  | (c) *Cohort study*—Summarise follow-up time (eg, average and total amount) Included in the Results on page 7. | |
| Outcome data | 15* | *Cohort study*—Report numbers of outcome events or summary measures over time | |
|  |  | *Case-control study—*Report numbers in each exposure category, or summary measures of exposure | |
|  |  | *Cross-sectional study—*Report numbers of outcome events or summary measures  Included in the Results on pages 7 and 8, and summarized in Tables 1 and 2. | |
| Main results | 16 | (*a*) Give unadjusted estimates and, if applicable, confounder-adjusted estimates and their precision (eg, 95% confidence interval). Make clear which confounders were adjusted for and why they were included  Included in the Results on pages 7, 8 and 9, and summarized in Tables 1 to 5 | |
|  |  | (*b*) Report category boundaries when continuous variables were categorized  Included in the Results on pages 7, 8 and 9, and summarized in Tables 1 to 5 | |
|  |  | (*c*) If relevant, consider translating estimates of relative risk into absolute risk for a meaningful time period. Not applicable. | |
| Other analyses | 17 | Report other analyses done—eg analyses of subgroups and interactions, and sensitivity analyses  Not applicable. | |
| Discussion | | | |
| Key results | 18 | Summarise key results with reference to study objectives  Included in the Discussion on page 9. | |
| Limitations | 19 | Discuss limitations of the study, taking into account sources of potential bias or imprecision. Discuss both direction and magnitude of any potential bias  Included in the Discussion on pages 12 and 13. | |
| Interpretation | 20 | Give a cautious overall interpretation of results considering objectives, limitations, multiplicity of analyses, results from similar studies, and other relevant evidence  Included in the Discussion on pages 9 to 13. | |
| Generalisability | 21 | Discuss the generalisability (external validity) of the study results  Included in the Discussion on page 13. | |
| Other information | | | |
| Funding | 22 | Give the source of funding and the role of the funders for the present study and, if applicable, for the original study on which the present article is based  Provided in the text on pages 7 and 13. | |
